# Radiology artificial intelligence, a systematic evaluation of methods (RAISE): a systematic review protocol

**DOI:** 10.1186/s13244-020-00929-9

**Published:** 2020-12-09

**Authors:** Brendan Kelly, Conor Judge, Stephanie M. Bollard, Simon M. Clifford, Gerard M. Healy, Kristen W. Yeom, Aonghus Lawlor, Ronan P. Killeen

**Affiliations:** 1 St Vincent’s University Hospital, Dublin, Ireland; 2 Insight Centre for Data Analytics, UCD, Dublin, Ireland; 3 Wellcome Trust – HRB, Irish Clinical Academic Training, Dublin, Ireland; 4 School of Medicine, University College Dublin, Dublin, Ireland; 5 HRB-Clinical Research Facility, NUI Galway, Galway, Ireland; 6 Plastic and Reconstructive Surgery, Mater Misicordiae University Hospital, Dublin, Ireland; 7 Lucille Packard Children’s Hospital at Stanford, Stanford, CA USA

**Keywords:** Radiology, Artificial intelligence, Systematic review, Methodology

## Abstract

**Introduction:**

There has been a recent explosion of research into the field of artificial intelligence as applied to clinical radiology with the advent of highly accurate computer vision technology. These studies, however, vary significantly in design and quality. While recent guidelines have been established to advise on ethics, data management and the potential directions of future research, systematic reviews of the entire field are lacking. We aim to investigate the use of artificial intelligence as applied to radiology, to identify the clinical questions being asked, which methodological approaches are applied to these questions and trends in use over time.

**Methods and analysis:**

We will follow the Preferred Reporting Items for Systematic Review and Meta-Analysis (PRISMA) guidelines and by the Cochrane Collaboration Handbook. We will perform a literature search through MEDLINE (Pubmed), and EMBASE, a detailed data extraction of trial characteristics and a narrative synthesis of the data. There will be no language restrictions. We will take a task-centred approach rather than focusing on modality or clinical subspecialty. Sub-group analysis will be performed by segmentation tasks, identification tasks, classification tasks, pegression/prediction tasks as well as a sub-analysis for paediatric patients.

**Ethics and dissemination:**

Ethical approval will not be required for this study, as data will be obtained from publicly available clinical trials. We will disseminate our results in a peer-reviewed publication.

*Registration number* PROSPERO: CRD42020154790

## Key Points

- This study presents a comprehensive methodology for a systematic review of the current state of Radiology Artificial Intelligence.
- Detailed characteristics of studies will be collected and analysed, including nature of task, disease, modality, subspecialty and data processing.
- Subgroup analysis will be performed to highlight differences in design characteristics between task, subspecialty modality and for trends in algorithm use over time.

## Introduction

### Background

There have been huge advancements in computer vision following the success of Deep Convolutional Neural Networks (CNN) at the 2012 ImageNet challenge [1]. Deep learning is a subset of machine learning, which itself is a subset of artificial intelligence (AI) the border field of how computers mimic human behaviour. The senior author of that seminal AlexNet paper, Geoffrey Hinton, advised in 2016 that we should stop training radiologists, as it was obvious that within 5 years deep learning (DL) would have surpassed them. While there have been major leaps forward in DL powered computer vision as it applies to radiology, the progress in performance has not yet materialised as he predicted. Rather, specific “narrow” applications have proven successful; and generalised superhuman performance remains elusive. Problems such as generalisability, stability and implementation, crucial in the medical field, have seen the clinical application of AI in healthcare lag behind other industries [2]. While recent guidelines have been established to advise on ethics, data management and the potential directions of future research [3–5], systematic reviews of the entire field are lacking. Our systematic review aims to look at the radiology AI literature from a task-specific point of view. Many of the roles of the clinical radiologist can be decomposed into tasks commonly faced by computer engineers in related computer vision fields such as segmentation, identification, classification and prediction [6].

### Objectives

This systematic review will aim to (1) assess the different methods and algorithms used to tackle these tasks, (2) to examine potential bias in methodology, (3) to consider the quality of data management in the literature and (4) outline trends in all the above.

## Methods and analysis

This systematic review has been registered with PROSPERO (registration number: CRD42020154790). We will report this systematic review according to the Preferred Reporting Items for Systematic Review and Meta-analysis (PRISMA) guidelines and have completed the PRISMA-P checklist for this protocol (Table 1).

**Table 1:** PRISMA-P (preferred reporting items for systematic review and meta-analysis protocols) 2015 checklist: recommended items to address in a systematic review protocol

Section and topic	Item nos.	Checklist item
*Administrative information*		
Title		
Identification	1a	Identify the report as a protocol of a systematic review (Title page)
Update	1b	If the protocol is for an update of a previous systematic review, identify as such (NA)
Registration	2	If registered, provide the name of the registry (such as PROSPERO) and registration number (Abstract)
Authors		
Contact	3a	Provide name, institutional affiliation, e-mail address of all protocol authors; provide physical mailing address of corresponding author (Title page)
Contributions	3b	Describe contributions of protocol authors and identify the guarantor of the review (title page)
Amendments	4	If the protocol represents an amendment of a previously completed or published protocol, identify as such and list changes; otherwise, state plan for documenting important protocol amendments (NA)
Support		
Sources	5a	Indicate sources of financial or other support for the review (Acknowledgments)
Sponsor	5b	Provide name for the review funder and/or sponsor (Acknowledgments)
Role of sponsor or funder	5c	Describe roles of funder(s), sponsor(s) and/or institution(s), if any, in developing the protocol (Acknowledgments)
Introduction		
Rationale	6	Describe the rationale for the review in the context of what is already known (introduction)
Objectives	7	Provide an explicit statement of the question(s) the review will address with reference to participants, interventions, comparators and outcomes (PICO) (objectives)
Methods		
Eligibility criteria	8	Specify the study characteristics (such as PICO, study design, setting, time frame) and report characteristics (such as years considered, language, publication status) to be used as criteria for eligibility for the review (methods/inclusion criteria)
Information sources	9	Describe all intended information sources (such as electronic databases, contact with study authors, trial registers or other grey literature sources) with planned dates of coverage (Methods/search methods)
Search strategy	10	Present draft of search strategy to be used for at least one electronic database, including planned limits, such that it could be repeated (Table 1 search methods)
Study records		
Data management	11a	Describe the mechanism(s) that will be used to manage records and data throughout the review (page 7 electronic search)
Selection process	11b	State the process that will be used for selecting studies (such as two independent reviewers) through each phase of the review (that is, screening, eligibility and inclusion in meta-analysis) (methods/selection and analysis)
Data collection process	11c	Describe planned method of extracting data from reports (such as piloting forms, done independently, in duplicate), any processes for obtaining and confirming data from investigators (methods/selection and analysis)
Data items	12	List and define all variables for which data will be sought (such as PICO items, funding sources), any pre-planned data assumptions and simplifications (methods/selection and analysis)
Outcomes and prioritisation	13	List and define all outcomes for which data will be sought, including prioritisation of main and additional outcomes, with rationale (methods/selection and analysis)
Risk of bias in individual studies	14	Describe anticipated methods for assessing risk of bias of individual studies, including whether this will be done at the outcome or study level, or both; state how this information will be used in data synthesis (methods/bias)
Data synthesis	15a	Describe criteria under which study data will be quantitatively synthesised (NA page methods-8)
	15b	If data are appropriate for quantitative synthesis, describe planned summary measures, methods of handling data and methods of combining data from studies, including any planned exploration of consistency (such as I^2^, Kendall’s τ)
	15c	Describe any proposed additional analyses (such as sensitivity or subgroup analyses, meta-regression)
	15d	If quantitative synthesis is not appropriate, describe the type of summary planned
Meta-bias(es)	16	Specify any planned assessment of meta-bias(es) (such as publication bias across studies, selective reporting within studies) (NA)
Confidence in cumulative evidence	17	Describe how the strength of the body of evidence will be assessed (such as GRADE) (Not included due to study heterogeneity)

### Inclusion/exclusion criteria for the selection of studies

#### Type of study design, participants

Two separate reviews are proposed, a primary review comprehensive of all literature and a secondary review in the paediatric literature only.

The comprehensive review will include all clinical radiological (not laboratory or phantom-based) deep learning papers that aim to complete a segmentation, identification, classification, or prediction task using computer vision techniques. Human hospital based studies that use computer vision techniques to aid in the care of patients through radiological diagnosis or intervention will be included. The paediatric review will include all machine learning and deep learning tasks as applied to paediatric clinical radiology.

#### Inclusion criteria

Clinical radiological papers that use DL computer vision techniques to complete a segmentation, identification, classification or prediction task based on radiographic, computed tomography (CT), magnetic resonance (MR), ultrasound (US) or nuclear medicine/molecular or hybrid imaging technique. Where the comparison group is a combined Human–AI performance, this will be specifically recorded.

#### Exclusion criteria

Functional MRI (fMRI) papers are not included as the techniques used in the computer analysis of fMRI data are quite separate from the computer vision-based tasks that are the subject of the review. To ensure focus on computer vision-based tasks and adequately assess these techniques, "radiomics" papers or those that focus on texture analysis or the identification of imaging biomarkers will be excluded from the primary review. Connectomics papers, quality assessment and decision support papers are not included. Image processing or registration papers are excluded. Image quality papers are excluded from the primary review. Papers solely for use in radiation therapy are also excluded. Non-human or phantom studies are excluded.

#### Type of intervention

We will not place a restriction on the intervention type and will include trials that study the clinical application of AI to radiology as outlined above.

### Search method for the identification of trials

#### Electronic search

We will perform electronic searches on MEDLINE (Pubmed), EMBASE from 2015 until 31 December 2019. Zotero will be used as our reference manager, and the Revtools package on R will be used to eliminate duplicate records. The search will be conducted in English. The search terms used are reported in Table 2. The artificial intelligence and radiology terms were combined with the AND operator with the addition of the paediatric terms with the AND operator for the paediatric sub-section. Search terms agreed by consensus between the two co-principle investigators with backgrounds in radiology and computer science respectively.

**Table 2:** Search terms

Artificial intelligence	Radiology	Paediatrics
(Artificial intelligence[Title/Abstract])OR (Machine learning[Title/Abstract])OR (Support vector machine[Title/Abstract])OR (SVM[Title/Abstract])OR (CNN[Title/Abstract])OR (RNN[Title/Abstract])OR (LSTM[Title/Abstract])OR (ResNet[Title/Abstract])OR (DenseNet[Title/Abstract])OR (Unet[Title/Abstract])OR (U-net[Title/Abstract])OR (DNN[Title/Abstract])OR (Neural network*[Title/Abstract])OR (Convolutional network*[Title/Abstract])OR (Deep learn*[Title/Abstract])OR (Semantic segmentation[Title/Abstract])OR (Ensemble[Title/Abstract])OR (Classification tree[Title/Abstract])OR (regression tree[Title/Abstract])OR (probability tree[Title/Abstract])OR (nearest neighbo*[Title/Abstract])OR (fuzzy logi*[Title/Abstract])OR (random forest[Title/Abstract])OR (kernel[Title/Abstract])OR (k-means[Title/Abstract])OR (naive bayes[Title/Abstract])	(X-ray*[Title/Abstract])OR (X-ray*[Title/Abstract])OR (Radiography[Title/Abstract])OR (Radiograph*[Title/Abstract])OR (Computed tomography[Title/Abstract])OR (CT[Title/Abstract])OR (CAT[Title/Abstract])OR (CTA[Title/Abstract])OR (Computerized axial tomography[Title/Abstract])OR (Magnetic resonance imag*[Title/Abstract])OR (MRI[Title/Abstract])OR (MR[Title/Abstract])OR (Magnetic resonance angio*[Title/Abstract])OR (MRA[Title/Abstract])OR (Scintigraphy[Title/Abstract])OR (DMSA[Title/Abstract])OR (Ultrasound*[Title/Abstract])OR (Sonograph*[Title/Abstract])OR (PET[Title/Abstract])OR (Positron Emission Tomography[Title/Abstract])OR (SPECT[Title/Abstract])OR (Single-photon emission[Title/Abstract])OR (Single photon emission[Title/Abstract])OR (mammogra*[Title/Abstract])	Infan* OR newborn* OR new-born* OR perinat* OR neonat* OR baby OR baby* OR babies OR toddler* OR minors OR minors* OR boy OR boys OR boyfriend OR boyhood OR girl* OR kid OR kids OR child OR child* OR children* OR schoolchild* OR schoolchild OR school child[tiab] OR school child*[tiab] OR adolescen* OR juvenil* OR youth* OR teen* OR under*age* OR pubescen* OR pediatrics[mh] OR pediatric* OR paediatric* OR peadiatric* OR school [tiab] OR school*[tiab] OR prematur* OR preterm*

#### Selection and analysis of trials

We will review the title and abstracts of studies to identify clinical radiological artificial intelligence studies for inclusion or exclusion. Studies with insufficient information to determine the use of AI computer vision methods will also be included for full-text review. We will then perform a full-text review to confirm studies that will be included in the final systematic review. This process will be summarised in a PRISMA flowchart. Abstract, title and full-text review will be performed by B.K. and S.B. Disagreements will be resolved by consensus or by a third reviewer (R.K.), if necessary.

Before full data extraction, all reviewers will complete the same 5% subsample and review answers to ensure there is a > 90% inter-reviewer agreement. Data extraction will be undertaken by three radiologists, two of whom are nationally certified and have a research interest in artificial intelligence (S.C. and G.H.). The third is a radiology resident in training with 4 years of experience who is a PhD candidate in radiology artificial intelligence.

Three reviewers will extract the following information in parallel and record in a custom database:

1. Country of origin (Paediatric Review only)
2. Radiology subspecialty
3. Retro/prospective
4. Supervised/unsupervised
5. Number of participants
6. Problem to be solved—i.e. segmentation, identification, classification, prediction.
7. Target Condition and body region
8. Reference Standard—Histology, rad report, surgery
9. Method for assessment of standard
10. Type of internal validation
11. External validation
12. Indicator method for predictor measurement, exclusion of poor-quality imaging Heatmap provided? Other explicability?
13. Algorithm—Architecture Transfer learning applied Ensemble architecture used
14. Data source—Number of images for training/tuning, Source of data, Data range, Open-access data
15. Was manual segmentation used?

Information will be extracted using a closed question format with an “add option” function if required. This is intended to maintain consistency while being flexible enough to account for the heterogeneity in the data. Please see Additional file 1. The full questionnaire will be made open access once the review is complete.

### Assessment of the quality of the studies: risk of bias

Due to the study design, there will be a high degree of heterogeneity within the study. This has been acknowledged in the literature to date [7]. We will, however, use basic surrogates of risk of bias including inclusion and exclusion criteria, internal or external validation and performance indication to estimate bias.

### Data synthesis

We will not perform a meta-analysis as part of this systematic review. A narrative synthesis of the data will be performed.

### Analysis by subgroups

We will report overall outcomes and outcomes by task, i.e. segmentation, identification, classification and prediction tasks. Descriptive statistics will be used to illustrate trends in the data.

### Study status

This systematic review will start in July 2020. We hope to have our first results in late 2020.

### Patient and public involvement

Our research group has engaged with a specific patient group MS Ireland to discuss their ideas, concerns and expectations around the clinical application of AI to radiology and these discussions continue to inform our research decisions.

### Ethics and dissemination

Ethical approval was not required for this study. We will publish the results of this systematic review in a peer-reviewed journal.

## Discussion

The volume of medical imaging investigations has greatly increased over recent years [8]. The number of clinicians trained in the expert interpretation of these investigations, however, has failed to keep pace in demand [9]. AI has been suggested as one possible solution to this supply/demand issue [8]. A huge volume of research has been published in a short time. Furthermore, the number of reviewers with expertise in both radiology and AI is limited. Standards for publication have only recently been developed [10]. This has the potential for papers of different levels of quality to be published and has the potential to negatively impact on patient care. Furthermore, many of the papers focus on a small range of pathology and tasks which opens the possibility of unnecessary duplication of work.

We anticipate that there will be rapid growth in the number of included papers year-on-year. We also expect that papers will be concentrated in a narrow range of topics. We aim to identify which algorithms are the most popular for particular tasks and also to investigate the presence of unique or custom models compared to off-the-shelf models. The issue of hyperparameter optimisation (whether automated or handcrafted) will also be examined. Statistical analysis will also be a feature of the review with a focus on sample size calculation and performance metrics [11].

We hope the systematic nature of this review will identify smaller papers with proper methods that may have been overlooked as well as highlight papers where some methods may have been suboptimal and provide an evidence base for a framework methodological design.

This review will have potential limitations, including publication and reporting bias. We will not be able to include studies with unpublished data, and we will misclassify studies that do not have clear reporting of adaptive designs in their methodology. Furthermore, the heterogeneity of the included studies will not allow for meaningful meta-analysis of results. The expected high number of included articles (in the range of 1000 articles over the 5 years 2015–2019) will only allow for a high-level overview of certain themes.

Finally, we hope to raise awareness of among the radiology community of the questions being asked as well as the methods being used to answer them with the radiology AI literature and give an overview of techniques for those with an engineering or computer science background looking to contribute to the field.

## Supplementary information

**Additional file 1**. Standard Data Extraction Questions.

## Abbreviations

AI: Artificial intelligence
CNN: Convolutional neural networks
CT: Computed tomography
DL: Deep learning
fMRI: Functional MRI
MR: Magnetic resonance
MS: Multiple sclerosis
PRISMA: Preferred reporting items for systematic review and meta-analysis
US: Ultrasound
