# Supplementary material for: Radiology artificial intelligence, a systematic evaluation of methods (RAISE): a systematic review protocol
Source: Insights Imaging. 2020 Dec 9;11:133. doi: 10.1186/s13244-020-00929-9 (PMC7726044; doi:10.1186/s13244-020-00929-9)
Supplement: Supplementary file 1 — Additional file 1. Standard Data Extraction Questions. [file 13244_2020_929_MOESM1_ESM.docx]

Section 1 of 2

Radiology AI Review General

Radiology AI review

DOI PMID or Title

*

Meets inclusion criteria

*

Yes

No

Needs Discussion

After section 1

Section 2 of 2

Untitled Section

Year

*

2015

2016

2017

2018

2019

2020

Task

*

Segmentation

Identification

Classification

Prediction

Time Series (RNN LSTM etc)

Generative

Change Detection

Other…

Model (The Primary model used or under investigation)

*

Not applicable

Custom (unique)

U Net

Inception

Ensemble (specify with other selections)

Alex Net

Res Net

Not Specified

RCNN

VGGNET

Shallow ANN

GAN

RNN

CNN-LSTM

holistically nested edge detector (HED)

DenseNet

Commercial (proprietary) deep learning system. Not further defined

Xception

GoogLeNet

SegNet

k-Nearest Neighbor (kNN)

back propagation (BP)

statistical expectation- maximization (EM)

Cascade Feed Forward Neural Network

RetinaNet

kernel fuzzy C-means algorithm with spatial information (SKFCM)

bidirectional chain coding method

BONENAVI version 2, involving three ANN systems

VCG-16

Learning-based multi-source IntegratioN frameworK for Segmentation of infant brain images (LINKS)

Adaboost

Locality-constrained Subcluster Representation Ensemble (LSRE) model

random forest (RF) regression

dynamic classifier selection-dynamic local training local tanimoto index (DCS-DLTLTI)

naïve Bayes (NB)

Deep belief network (DBN)

support vector machine (SVM)

Overfeat (pre-trained convolutional neural network)

discriminant Grassmannian manifold

Generalized Linear Models

Other…

Any additional models used or investigated

*

Not applicable

Custom (unique)

U Net

Inception

Ensemble (specify with other selections)

Alex Net

Res Net

Not Specified

RCNN

VGGNET

Shallow ANN

GAN

RNN

CNN-LSTM

They tested modifications of the HED network

DenseNet

Principal Component Discriminant Analysis (PCDA)

support vector machine (SVM)

MAGIC 5

GrowCut (IGC) algorithm.

Backpropagation neural network

BONENAVI version 1

Radon transform

articulated statistical shape model (aSSM)

Convolutional neural network (CNN)

a hidden Markov random field model with maximum entropy (HMRF- EM)

artificial immune system (AIS)

Hidden Markov Model (HMM)

Gradient Boosting classifier (GB)

Other…

If Previously described algorithm (e.g. UNet) used was it modified or "off the shelf"?

*

Not applicable

Modified

Off the Shelf

Commercial (proprietary) deep learning system. Not further defined

UNKNOWN

Other…

Design Time

*

Prospective

Retrospective

Not applicable

Unclear

Other…

Type of study

*

Randomised controlled trial

Cohort studie

Case-control studies

Cross-sectional studies

Qualitative studies (unlikely but this option is included for completeness)

Other…

Supervised

*

Supervised

Unsupervised

Not applicable

unclear

Other…

Transfer Learning

*

Yes

No

Not applicable

Other…

Modality

*

CT

MR

Plain Film

US

Nuclear Med/ Molecular/ Hybrid

Interventional Radiology

PET-CT

Mammography

Other…

Subspecialty

*

Neuro

Head and Neck

Chest

Cardiac and vascular

Abdominal and GI

Obs/Gynae

Breast

MSK

Paeds

Cancer Imaging

Emergency Rad

Nuclear

Interventional

Urogenital

Other…

Disease (Try to be general here to avoid having too many categories)

*

Not applicable

Normal Patients or No specific disease

Lung Nodules

Alzheimers

Cancer Imaging

Multiple Sclerosis

Prognosis

Bone Age

Haemorrhage

CAD

prostate cancer

lung cancer

pneumonia

Breast Cancer

Knee injury

pneumothorax

Thoracic disease

MSK pathology / trauma

Stroke

Other…

Reference standard

*

Rad Report

Pathology

Other Specialist Report

Not applicable

An expert panel combined the results of the DL system under investigation and double reading to form the reference standard

panel of experts

unclear

Other…

Evaluation Metric

*

Dice

AUC

Sens/Spec

Precision/Recall

Not applicable

3-class weighted average F1 score

F1

accuracy

Kappa index

correlation

re-inclusion ratio

recognition rate

Averaged validation accuracy

confusion matrix

overlap score

performance index

determination of corresponding ratio (CR) and percent match (PM )

positive/ negative predictive value

Hausdorff distance (HD)

Other…

Performance

(Internal or external validation (ie are the results validated on another hospitals images)

*

Internal

External

Not applicable

Study included a cases from 6 different institutions, however the training, validation and test sets contained a mixture of cases from the 6 institutions

Public data set

none

Other…

Data Augmentation

*

Simple (symmetry operations)

Generation (eg with GANs)

Not applicable

None

Other…

Explicability (Why the AI came to a decision)

*

Visualisation (eg Heat map)

Analytic/Didactic (natural language explanation)

Cases/examples

Rejections of alternatives (ie chosen as other options removed as less likely)

Counterfactual example (e.g. "if Oswald didnt kill JFK someone else did")

Not applicable

None

Other…

Were (Hyper)parameters Optimized

*

Yes

No

Unclear

Other…

Data Source

*

One Hospital

More than One Hospital

Public Data Set

Public and local data combined

Not applicable

Unclear

Other…

Mention a Power analysis?

*

YES

No

Not applicable

Other…

Explain inclusion criteria

*

YES

No

Not applicable

Other…

Number of Patients

Number of Cases

Number of Images

Manual Segmentation/Data Processing

*

Yes

No

Not applicable

Other…

Model/Code Open access?

*

Yes

No

Not applicable

Other…

Data Open access?

*

Yes

No

Not applicable

Other…

Performance Comparison

*

Radiologists

State of the art Model

None

Not applicable

Panel of experts including rad + other specialties

Other machine learning models

Other…

Is "change over time" mentioned or is this addressed a "time series" problem?

*

Yes

No

Not applicable

Other…

Data Collection Compete

*

Yes

No

Other…
